# Upregulation of GOLPH3 mediated by Bisphenol a promotes colorectal cancer proliferation and migration: evidence based on integrated analysis

**DOI:** 10.3389/fphar.2024.1337883

**Published:** 2024-05-17

**Authors:** Lihua Chen, Shaojian Chen, Yachen Li, Yi Qiu, Xiaojing Chen, Yuze Wu, Xian Deng, Mingliang Chen, Chunxiao Wang, Zhongshi Hong, Chengzhi Qiu

**Affiliations:** ^1^ Department of General Surgery, The Second Affiliated Hospital of Fujian Medical University, Quanzhou, China; ^2^ The 2nd Clinical College of Fujian Medical University, Quanzhou, China; ^3^ Medical Department of the Second Affiliated Hospital of Fujian Medical University, Quanzhou, China

**Keywords:** bisphenol A, GOLPH3, colorectal cancer, endocrine-disrupting chemicals, cancer genomics, tumor microenvironment, oncogenic pathways

## Abstract

**Background:**

The interaction between environmental endocrine-disrupting chemicals, such as Bisphenol A (BPA), and their influence on cancer progression, particularly regarding the GOLPH3 gene in colorectal cancer, remains unclear.

**Methods:**

We performed an integrated analysis of transcriptional profiling, clinical data, and bioinformatics analyses utilizing data from the Comparative Toxicogenomics Database and The Cancer Genome Atlas. The study employed ClueGO, Gene Set Enrichment Analysis, and Gene Set Variation Analysis for functional enrichment analysis, alongside experimental assays to examine the effects of BPA exposure on colorectal cancer cell lines, focusing on GOLPH3 expression and its implications for cancer progression.

**Results:**

Our findings demonstrated that BPA exposure significantly promoted the progression of colorectal cancer by upregulating GOLPH3, which in turn enhanced the malignant phenotype of colorectal cancer cells. Comparative analysis revealed elevated GOLPH3 protein levels in cancerous tissues *versus* normal tissues, with single-cell analysis indicating widespread GOLPH3 presence across various cell types in the cancer microenvironment. GOLPH3 was also associated with multiple carcinogenic pathways, including the G2M checkpoint. Furthermore, our investigation into the colorectal cancer microenvironment and genomic mutation signature underscored the oncogenic potential of GOLPH3, exacerbated by BPA exposure.

**Conclusion:**

This study provides novel insights into the complex interactions between BPA exposure and GOLPH3 in the context of colorectal cancer, emphasizing the need for heightened awareness and measures to mitigate BPA exposure risks. Our findings advocate for further research to validate these observations in clinical and epidemiological settings and explore potential therapeutic targets within these pathways.

## Introduction

Endocrine-disrupting chemicals (EDCs) represent a significant environmental concern with global health implications (Yilmaz et al., 2020; Kiess and Haeusler, 2021). Found in various consumer products like plastics, pesticides, and personal care items, EDCs can mimic or interfere with the body’s natural hormone signals (Ribeiro et al., 2017). As our understanding of how EDCs exert their influence grows, the need for a deeper understanding of their impact on cancers becomes increasingly clear (Buoso et al., 2020a; Masi et al., 2021). Colorectal cancer, a common type of gastrointestinal tumor, is a major contributor to global cancer mortality rates (Labianca et al., 2010). The past few years have witnessed a rise in its occurrence, possibly linked to improvements in living conditions and changes in daily routines (Auclin et al., 2017). Early symptoms of colorectal cancer often go unnoticed, leading to late-stage diagnosis and complex treatment processes (Holtedahl et al., 2021). In addition to factors like genetics, dietary patterns, and broader environmental influences, EDCs also play a role in triggering colorectal cancer (Pham et al., 2020). Research suggests that regular health screenings and adopting healthier lifestyles can significantly reduce the risk of this disease. Early detection and treatment remain critical for improving patient survival rates.

Bisphenol A (BPA) is a widely used chemical compound in the manufacturing of plastics and resins (Michałowicz, 2014). In recent years, several investigations have concentrated on the associations between BPA and a range of health concerns, especially its endocrine-disrupting properties (Rochester, 2013). Research indicates that BPA can mimic endogenous estrogen, potentially leading to hormonal imbalances. Such imbalances are believed to be linked to the onset and progression of diseases like breast cancer, prostate cancer, and other malignancies (Di Donato et al., 2017; Hafezi and Abdel-Rahman, 2019; Liu et al., 2021). Furthermore, BPA exposure has been linked to reproductive issues, neurodevelopmental problems, and cardiovascular diseases, among other health risks (Cimmino et al., 2020). Studies have shed light on the mechanisms by which BPA promotes colon cancer development. Xia et al. (Xia et al., 2022) identified that BPA accelerates cancer progression by concurrently targeting the NADPH oxidase and the mitochondrial electron transport chain, leading to ROS production and activation of the HIF-1α/VEGF/PI3K/AKT pathway. Additionally, Jun et al. (Jun et al., 2021) revealed that BPA exposure significantly enhances the proliferation, migration, and tumor growth in human colon cancer cells through ERK pathway activation, E-cadherin expression reduction, and 5-HT3 receptors upregulation, suggesting a potential link between BPA exposure and colon cancer development. Consequently, understanding the potential impacts of BPA on human health and taking measures to minimize exposure risks have become major areas of focus in both public and scientific domains. Current evidence suggests that the GOLPH3 gene encodes a protein with a pivotal role in the Golgi apparatus, contributing to vesicular trafficking and phosphatidylinositol 4-phosphate signaling (Kuna and Field, 2019). It has become a research hotspot given its oncogenic potential, as it is often overexpressed in various cancers, leading to enhanced tumor growth, progression, and poor prognosis (Giansanti and Piergentili, 2022). Specifically, in colon cancer, GOLPH3 overexpression has been linked to tumor aggressiveness, metastasis, and resistance to therapy. Supporting this notion, (Yu et al., 2020), Yu et al. demonstrated that inhibition of GOLPH3 overcomes oxaliplatin resistance in colon cancer cells through downregulation of the PI3K/AKT/mTOR signaling pathway. Additionally, Zhang et al. (Hong et al., 2020) identified miR-3150b-3p as a suppressor of colorectal cancer cell progression by targeting GOLPH3. Given the known impacts of BPA on pathways similar to those influenced by GOLPH3, such as the PI3K/AKT pathway, a potential link between BPA exposure and GOLPH3 modulation in cancer warrants further investigation. Also, the evidence regarding BPA effect on GOLPH3 expression is still conflicting (Ali et al., 2014; Schaffert et al., 2021). Therefore, the exploration of this connection could provide a novel perspective on how environmental factors like BPA contribute to cancer pathogenesis through molecular mechanisms involving key regulatory proteins like GOLPH3.

Our findings demonstrated that BPA exposure accelerates colon cancer progression by upregulating GOLPH3 expression, consequently exacerbating the malignant characteristics of cancer cells.

## Methods

### Data collection

Relevant information on BPA and its relationship with diseases was obtained from the Comparative Toxicogenomics Database (CTD) (Davis et al., 2023). Transcriptional profiling data and medical records of individuals with colon cancer were obtained from The Cancer Genome Atlas (TCGA) database. We used R language to acquire the STAR-Counts data from the TCGA-GDC portal and extracted transcriptome data in the Transcripts Per Kilobase Million (TPM) format. Annotation for ENSM ID was conducted using the genomic annotation file. Gene expression data were normalized before analysis to improve data quality. Clinical data were downloaded directly from the TCGA-GDC portal and organized and summarized using a Perl script.

### Biological relation analysis

We explored biological relationships using clueGO, Gene Set Enrichment Analysis (GSEA), and Gene Set Variation Analysis analysis (Subramanian et al., 2005; Bindea et al., 2009; Hänzelmann et al., 2013). For the GSEA analysis, we employed Hallmark, Gene Ontology (GO) and Kyoto Encyclopedia of Genes and Genomes (KEGG) gene sets as reference gene sets (Kanehisa and Goto, 2000; The Gene Ontology Consortium, 2019).

### Data from the human protein atlas (HPA) database

The HPA database, which records protein expression across various cells, tissues and organs provided direct protein levels of GOLPH3 in both normal and colon cancer tissues (Uhlén et al., 2015). We accessed the HPA database and searched for GOLPH3 by entering the protein name into the search bar. The search results were divided into two categories: “Tissue” for normal tissues, where we specifically looked at the liver, and “Pathology” for cancerous tissues, where liver cancer was examined. Visualization of GOLPH3 expression in these tissues was achieved by downloading images directly from the search results.

### Single-cell analysis

We utilized the Tumor Immune Single-cell Hub website (Sun et al., 2021) to perform a single-cell analysis. Following established protocols, we queried the database by entering the gene of interest into the search bar. This facilitated the characterization of GOLPH3 expression at the single-cell level across diverse cancer types.

### Tumor microenvironment analysis

We quantified the tumor microenvironment of colon cancer patients using the gene expression profile profiles as input, along with multiple algorithms, including MCPCOUNTER, QUANTISEQ, EPIC, CIBERSORT and TIMER, we quantified the tumor microenvironment of colon cancer (Becht et al., 2016; Li et al., 2017; Chen et al., 2018; Plattner et al., 2020; Racle and Gfeller, 2020). Notably, each algorithm primarily utilizes the transcription profile data as input.

### Tumor mutation analysis

We examined the genetic alterations within cancer cells by evaluating two critical metrics: Tumor Mutational Burden (TMB) and Microsatellite Instability (MSI). TMB was calculated by dividing the total number of nonsynonymous mutations by the size of the coding region of the genome analyzed, expressed as mutations per megabase (mut/Mb). MSI was assessed based on the number and length of microsatellite regions that exhibit instability.

### Drug sensitivity analysis

Data regarding the sensitivity of particular drugs was sourced from the Genomics of Drug Sensitivity in Cancer (GDSC) database (Yang et al., 2013). The IC50 values for these drugs are determined by aligning information from the GDSC database with that of the TCGA database, providing relative measurements of drug efficacy.

### Plasmid acquisition and construction

To generate the pGL2-GOLPH3Promoter-luc (pGP3-WT-luc) reporter plasmid containing the wild-type sequence, a 488 base pair segment spanning from −254 to +234 of the human GOLPH3 promoter was cloned. This segment was amplified from human genomic DNA through polymerase chain reaction (PCR). The primers used for PCR were: pGP3-WT, forward, 5′-TGC AGA​CGC​GTC​CCA​GGC​TCT​TCC​ATT​CAC-3′, reverse, 5′-TGCAGAAGCTTRCCGGGTTTCCGTGTTAAAT-3’. The site-directed mutagenesis plasmids pGP3-E2Fm and pGP3-CREBm were purchased from the MiaoLingPlasmid (China).

### Detection of luciferase activity

In experiments using SW480 cells for measuring luciferase activity, cells were initially seeded in six-well plates at a density of 150,000 cells per well. After 24 h, cells underwent co-transfection with 500 ng of a firefly luciferase reporting vector and 20 ng of a Renilla luciferase control vector (pRL-TK). The total DNA quantity used per reaction was adjusted to 1 µg with the pCMV vector. Luciferase activity was then assessed 24 h post-transfection using the Dual-Luciferase Reporter Assay System from Promega, based in Madison, WI, United States, following the protocols provided. Results were adjusted for transfection efficiency based on the Renilla luciferase activity in each sample, reporting the results in Relative Luciferase Units (RLU). For baseline comparison, the luciferase activity of samples transfected only with the reporter vector was used.

### Cell lines and BPA exposure

Human colorectal adenocarcinoma cell lines SW480 and DLD-1 were routinely cultured in Dulbecco’s Modified Eagle Medium (DMEM) supplemented with fetal bovine serum (FBS) and 1% penicillin-streptomycin. The human monocyte cell line THP-1 and the normal colon mucosal cell line NCM460 were cultured in RPMI-1640 medium supplemented with 10% fetal bovine serum, 0.05 mol/L 2-mercaptoethanol, 100 U/mL penicillin, and 100 μg/mL streptomycin. All cell lines were maintained in a humidified incubator at 37°C with 5% CO_2_. Upon reaching 70%–80% confluence, cells were passaged using a 0.25% trypsin-EDTA solution. Cultures were regularly monitored to ensure *mycoplasma* absence. BPA (B802575, Macklin Biochemical Co., Ltd., Shanghai, China) was prepared as a 1 M stock solution in dimethyl sulfoxide (DMSO, Minibio, China). Working solutions at various concentrations were then prepared from the stock. The experimental design included a control group cultured only in a complete medium and treatment groups exposed to 1 μM BPA for 24 h.

### Real-time quantitative PCR (qPCR) and cell transfection

For qPCR analysis, the following commercially available kits, equipment, and primers were used: Total RNA was extracted from cells using TRIzol reagent (Invitrogen, United States). A reverse transcription kit (Thermo Fisher Scientific, United States) was then employed to synthesize complementary DNA (cDNA) from 1 μg of total RNA following the manufacturer’s instructions. The amplification process was conducted using the SYBR Green system (Thermo Fisher Scientific, United States), with gene expression levels normalized to the housekeeping gene GAPDH. Quantitative analysis of gene expression was performed using the 2^−ΔΔCT^ method. Regarding cell transfection, the procedure was as follows. Cells were plated in six-well plates and allowed to grow until reaching 60%–80% confluence. Transfection was then performed using Lipofectamine 3,000 (Thermo Fisher Scientific, United States) according to the manufacturer’s protocol. Briefly, the shRNA plasmid targeting GOLPH3 (Tsingke Biotechnology Co., Ltd) was combined with the transfection agent and serum-free medium. After a 20-min incubation at room temperature, the mixture was added to the cells. Following a specified incubation period, the transfection medium was replaced with a standard growth medium, and the cells were incubated for further experimentation. The primer used for qPCR were: GOLPH3, forward, 5′-AGG​GCG​ACT​CCA​AGG​AAA-3′, reverse, 5′-TGA​TGT​GTA​ACC​CTC​GCG-3’; GAPDH, forward, 5′-GGT​CAT​AAG​CTT​GCG​TTG​ATT​AAG-3′, reverse, 5′-CTA​CGG​AAA​CCT​TGT​TAC​GAC​TTT-3’. The target sequence of shRNA of GOLPH3 was: shRNA#1, CCG​CCT​TAC​TCT​TAT​GGA​AGA, shRNA#2, CGC​AAA​GAA​CCT​AGT​AGA​GAA, shRNA#3, CGA​CTA​CTA​GAC​AGA​AAG​GTA.

### Western blot

To investigate the impact of BPA exposure on GOLPH3 protein levels, Western blot analysis was performed. Following the treatment of SW480 and DLD-1 cell lines with or without BPA exposure, cells were lysed using RIPA buffer (Beyotime Biotechnology, China) supplemented with a protease inhibitor cocktail (Sigma-Aldrich, United States) to extract total protein. Protein concentration was determined using the BCA Protein Assay Kit (Thermo Fisher Scientific, United States) according to the manufacturer’s instructions. Equal amounts of protein (30 μg) were separated by SDS-PAGE on a 12.5% gel and subsequently transferred onto PVDF membranes (Millipore, United States). The membranes were blocked with 5% non-fat milk in TBST (Tris-buffered saline, 0.1% Tween 20) for 1 h at room temperature to prevent non-specific binding. The membranes were then incubated overnight at 4°C with primary antibodies against GOLPH3 (1:1,000, Abcam, United States) and GAPDH (1:5,000, Abcam, United States) as a loading control. After washing with TBST, the membranes were incubated with HRP-conjugated secondary antibodies (1:2000, Cell Signaling Technology, United States) for 1 h at room temperature. Protein bands were visualized using the ECL Western blotting Detection Reagent (GE Healthcare, United States) and captured with a chemiluminescence imaging system (Bio-Rad, United States).

### Cell proliferation assays

Cell proliferation was assessed using colony formation and CCK-8 assays. For the CCK-8 assay, cells were seeded at a density of 3,000 cells per well in 96-well plates and allowed to adhere during incubation. At specified time intervals (0, 24, 48, and 72 h), 10 μL of CCK-8 solution were added to each well and incubated for 2 h at 37°C. A microplate reader was then used to measure the absorbance at 450 nm. Cell proliferation rates were calculated using optical density measurements. For the colony formation assay, 500 cells were seeded in each well of 6-well plates and allowed to proliferate for 10 days with media changes every 3–4 days. After observable colonies formed, cells were rinsed with phosphate-buffered saline (PBS), fixed with 4% paraformaldehyde for 15 min, and stained with 0.1% crystal violet for 30 min.

### Macrophage induction from monocytes and flow cytometry

THP1 cells, cultured in six-well plates, were treated with 100 ng/mL of phorbol-12-myristate-13-acetate (PMA; Sigma-Aldrich) and incubated for 24–48 h. After incubation, the medium was replaced with fresh PMA-free medium, and the cells were maintained for another 3 days before use. For the detection of surface markers, cells in chilled phosphate-buffered saline (PBS) were treated with either anti-CD206 or anti-HLA-DR antibodies (both from eBioscience) at 4°C for 30 min. Following incubation, the cells were washed and subsequently analyzed using a BD Accuri™ C6 flow cytometer (BD Biosciences, San Jose, CA, United States) to detect CD206 and HLA-DR.

### Apoptosis detection

To evaluate the pro-apoptotic effect of BPA exposure in colon cancer cells, apoptosis was assessed using Annexin V-FITC/PI double staining flow cytometry analysis. For this assay, SW480 and DLD-1 cell lines treated with and without BPA, as described in the cell lines and BPA exposure section, were collected by trypsinization and washed twice with cold PBS. The cells were then resuspended in 1X binding buffer at a concentration of 1 × 10^6^ cells/mL. Subsequently, 100 μL of the suspension (approximately 1 × 10^5^ cells) was transferred to a 5 mL culture tube, to which 5 μL of Annexin V-FITC and 5 μL of propidium iodide (PI) (Annexin V-FITC Apoptosis Detection Kit, BD Biosciences, United States) were added. The cells were gently vortexed and incubated for 15 min at room temperature in the dark. After incubation, 400 μL of 1X binding buffer was added to each tube. Flow cytometry was performed using a BD FACSCanto™ II system (BD Biosciences, United States), and the data were analyzed with BD FACSDiva™ software. Cells were classified into live (Annexin V−/PI−), early apoptotic (Annexin V+/PI−), late apoptotic (Annexin V+/PI+), and necrotic (Annexin V−/PI+) populations. A minimum of 10,000 events were recorded for each sample. The percentage of apoptotic cells was calculated by summing the early and late apoptotic cell populations.

### Transwell assay

To prepare the cells for the assay and ensure a synchronized and quiescent cell cycle, they were serum-starved for 24 h. Transwell inserts with an 8.0 μm pore size (compatible with a 24-well plate) were used for both the migration and invasion assays. For the invasion assay, the upper surface of the inserts was coated with Matrigel (Corning, United States) to simulate the extracellular matrix barrier. The lower chamber was filled with a medium containing 10% FBS to act as a chemoattractant. Concurrently, one hundred thousand cells, suspended in serum-free medium, were seeded into the upper chamber of each insert. The assembly was then incubated at 37°C in a humidified atmosphere with 5% CO_2_ for 24 h, promoting cell migration through the membrane pores and invasion through the Matrigel barrier. Post-incubation, non-migratory or non-invasive cells on the upper surface of the membrane were gently removed with a cotton swab. Cells that had migrated or invaded the underside were fixed with 4% paraformaldehyde for 15 min and stained with 0.1% crystal violet for 30 min. The migrated and invaded cells were quantified by counting them in several random microscopic fields.

### Wound-healing assay

To evaluate how BPA exposure and GOLPH3 modulation affect colorectal cancer cell migration, a wound-healing assay was performed. SW480 and DLD-1 cells were seeded in 6-well plates and grown to near confluence (90%–100%). To minimize the influence of proliferation, the cells were serum-starved for 24 h before a sterile pipette tip scratched a straight line across the cell monolayer, creating a “wound”. The wells were then washed with PBS to remove detached cells and debris, and fresh DMEM containing either control medium or specified BPA concentrations was added. Finally, images of the wound were captured at 0 h (immediately after scratching) and again at 12 or 24 h using an inverted microscope with a digital camera.

### Statistical analysis

The mean ± standard deviation of all data was reported based on a minimum of three independent experiments. The chi-squared test or Fisher’s exact test was used to analyze categorical data, as deemed appropriate. The Kaplan-Meier method was utilized to plot survival curves and compared using the log-rank test. A *p*-value below 0.05 was statistically significant. Statistical software (SPSS, GraphPad Prism, and R software) was utilized for all statistical analyses and graph creation.

## Results

The overall workflow of the study is depicted in a flowchart in Supplementary Figure S1.

### GOLPH3 promotes the proliferation and migration ability of colon cancer cell

First, we investigated the role of GOLPH3 in colon cancer. qRT-PCR analysis (Supplementary Figure S2A) revealed upregulated GOLPH3 mRNA levels in both SW480 and DLD-1 cells. Next, we knocked down GOLPH3 to assess its impact on colon cancer cell behavior. shRNA#2 displayed the most efficient knockdown, as evidenced by qPCR results (Supplementary Figure S2B, C). Inhibition of GOLPH3 significantly suppressed the proliferation ability of colon cancer cells, as demonstrated by CCK8 and colony formation assays (Supplementary Figure S2D–F). The transwell assay (Supplementary Figure S2G) further indicated that GOLPH3 suppression effectively hampered the invasion and migration capacity of colon cancer cells. The wound-healing assay (Supplementary Figure S2H) supported this finding, showing reduced migration ability in cells with GOLPH3 knockdown. To verify our results, we overexpressed GOLPH3 in SW480 cells. Overexpression of GOLPH3 in SW480 cells, confirmed by qRT-PCR (Supplementary Figure S3A), significantly enhanced the proliferation ability as shown by CCK8 and colony formation assays (Supplementary Figure S3B, C). Transwell and wound-healing assays (Supplementary Figure S3D, E) indicated that overexpression of GOLPH3 could significantly enhance the invasion and migration ability of SW480 cells.

### Colon cancer exhibits a distinct expression pattern of GOLPH3

We next investigated the expression pattern of GOLPH3 in colon cancer by analyzing data from various open-access databases containing information on colon cancer patients. While high expression of GOLPH3 has been observed in various organs, suggesting its biological importance (Supplementary Figure S4A), no significant difference in GOLPH3 RNA expression was detected between healthy and colon cancer tissue (Supplementary Figure S4B, C). Interestingly, our data revealed upregulated GOLPH3 protein levels in colon cancer tissue compared to normal intestinal epithelium (Supplementary Figure S4D, E). This finding suggests a potential post-transcriptional regulatory mechanism affecting GOLPH3 expression in colon cancer and normal intestinal epithelium (Supplementary Figure S4D, E). Furthermore, single-cell analysis of data from multiple public databases, including EMTAB8107, GSE108989, GSE139555, GSE146771_10X, GSE136394, and GSE146771, demonstrated GOLPH3 expression across most cell types within the colon cancer microenvironment (Supplementary Figure S4F–K).

### Biological enrichment analysis

**FIGURE 1:**
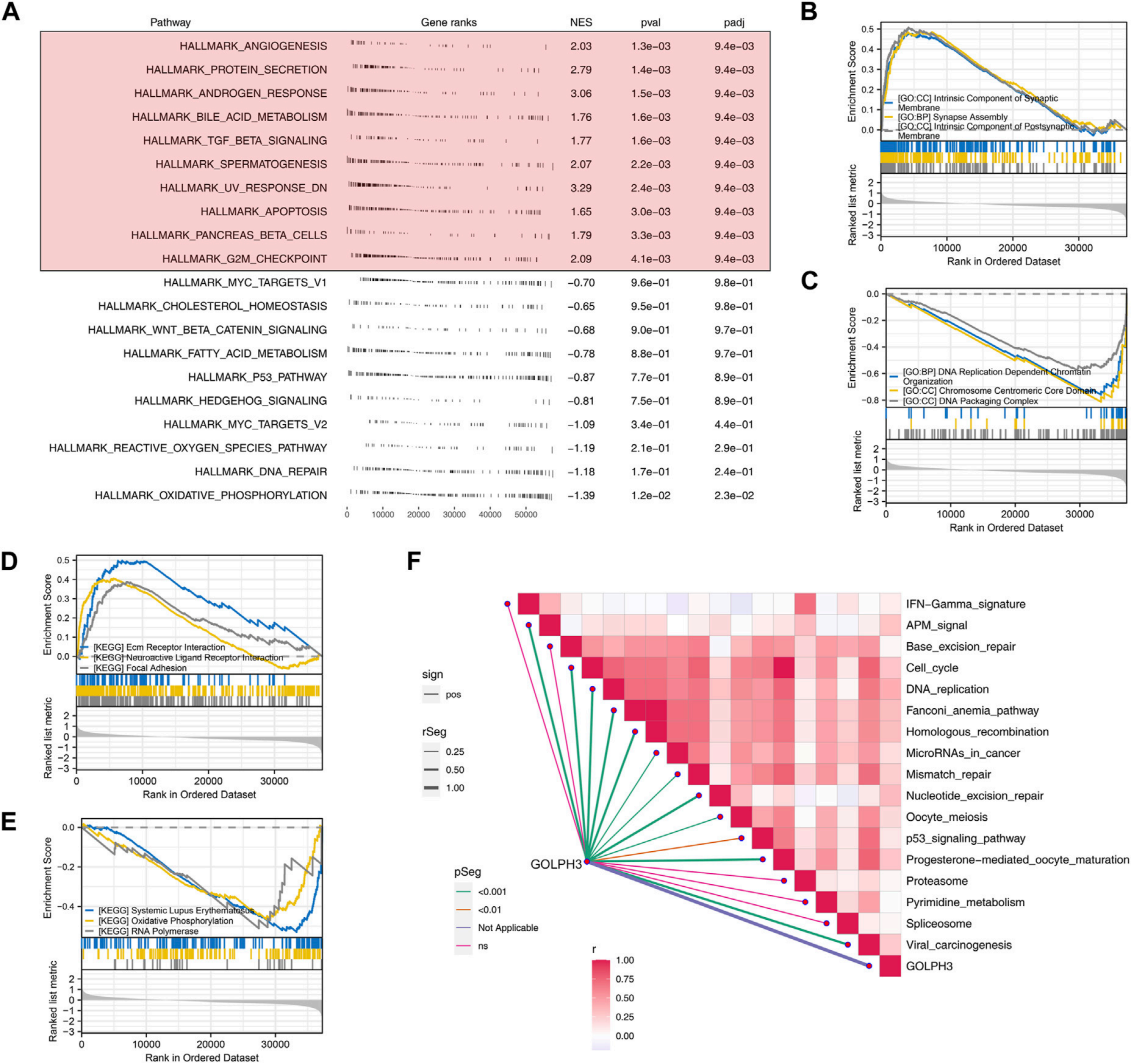
GOLPH3 enrichment analysis highlights its involvement in key biological processes and pathways in colon cancer. Notes: **(A)** GSEA reveals significant enrichment of GOLPH3 in related based on Hallmark gene set. Enrichment plots highlight the differential activation of these pathways in samples with high *versus* low GOLPH3 expression, using nominal *p*-values and normalized enrichment scores (NES) to determine statistical significance; **(B)** The top three upregulated GO terms identified through GSEA for high GOLPH3 expression in colon cancer; **(C)** The top three downregulated GO terms identified through GSEA for high GOLPH3 expression in colon cancer; **(D)** The top three upregulated KEGG terms identified through GSEA for high GOLPH3 expression in colon cancer; **(E)** The top three downregulated KEGG terms identified through GSEA for high GOLPH3 expression in colon cancer; **(F)** This panel presents a comprehensive correlation analysis between GOLPH3 expression levels and the activation of various immune pathways within the colon cancer microenvironment (Spearman’s correlation analysis).

To investigate the biological functions of GOLPH3 in colorectal cancer and its potential modulation by BPA exposure, we employed GSEA with the Hallmark gene set. This analysis revealed a significant association of GOLPH3 with processes critical for tumor development and progression, potentially influenced by BPA (Figure 1A). These processes included angiogenesis (tumor growth and metastasis), protein secretion (cell communication and signaling), androgen response (hormone-driven cancers), and bile acid metabolism (relevant to colorectal cancer). Interestingly, processes like TGF-β signaling (known for its dual role in cancer progression and suppression), spermatogenesis (cellular differentiation), UV response (DNA damage repair), apoptosis (cancer cell survival), pancreatic beta cell function (metabolic dysregulation), and the G2M checkpoint (cell cycle regulation) were also highlighted. Further analysis using GO and KEGG pathways provided deeper insights into GOLPH3’s role. The most upregulated GO terms emphasized the importance of synaptic components and assembly, suggesting a role for GOLPH3 in cellular communication and neuronal-like signaling in cancer, given BPA exerts well-established endocrine-disrupting effects on similar pathways. Conversely, the downregulated GO terms focused on DNA organization and replication, implying a potential mechanism for GOLPH3’s influence on genomic stability (Figures 1B,C). KEGG pathway analysis revealed upregulation in focal adhesion and neuroactive ligand-receptor interaction, indicating enhanced cell adhesion and signaling under GOLPH3 influence. Downregulation was observed in pathways related to systemic immune responses (lupus), oxidative phosphorylation, and RNA polymerase activity, suggesting a complex interplay between cellular energy metabolism, transcriptional control, and possibly immune evasion (Figures 1D,E). Immune correlation analysis identified a positive correlation between GOLPH3 and various processes, including cell_cycle, DNA_replication, fanconi_anemia_pathway, homologous_recombination, microRNAs_in_cancer, mismatch_repair, nucleotide_excision_repair, oocyte_meiosis, P53_signaling_pathway, progesterone-mediated_oocyte_maturation and viral_carcinogenesis (Figure 1F).

### GOLPH3 is associated with genomic instability

**FIGURE 2:**
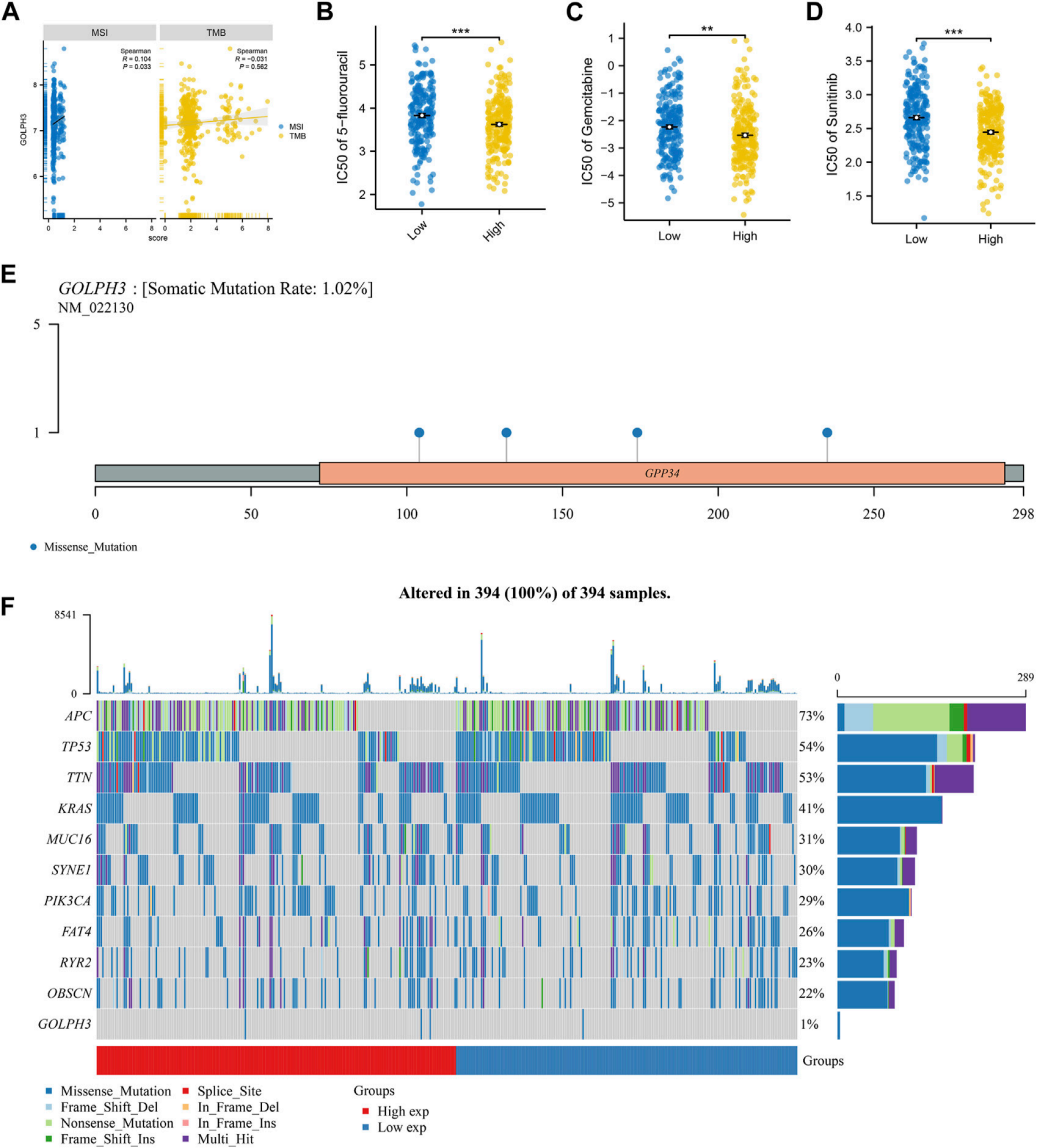
Association of GOLPH3 with genomic instability and drug sensitivity in colon cancer. Notes: **(A)** This panel illustrates the correlation between GOLPH3 expression levels and genomic instability scores (TMB and MSI) across colon cancer samples. The correlation coefficient and corresponding statistical significance are derived from Spearman’s rank correlation analysis; B–D: Drug sensitivity analysis based on GOLPH3 expression levels, showcasing the differential response to chemotherapy agents: 5-fluorouracil **(B)**, gemcitabine **(C)**, and sunitinib **(D)** in colon cancer patients with high *versus* low GOLPH3 expression. Sensitivity to these drugs was quantified using IC50 values, with lower IC50 values indicating higher sensitivity. Statistical significance was evaluated using the Mann-Whitney U test, comparing high and low GOLPH3 expression groups, ** = *p* < 0.01, *** = *p* < 0.001; E–F: Mutation landscape of GOLPH3 in colon cancer, highlighting the frequency and distribution of mutations within the GOLPH3 gene across a cohort of colon cancer patients. **(E)** shows a mutation frequency plot, while **(F)** provides a detailed view of mutation types and their locations within the gene.

It is now understood that genomic instability can also affect tumor progression. We next investigated the association between GOLPH3 expression and genomic instability in colon cancer patients. TMB is defined as the total number of mutations per coding area of a tumor genome, serving as an indicator of neoantigen load. A higher TMB is often associated with better responses to immunotherapy, as it may increase the likelihood of developing neoantigens that are recognizable by the immune system. Conversely, MSI is a condition of genetic hypermutability that results from impaired DNA mismatch repair (MMR) systems, leading to the accumulation of insertion-deletion mutations at microsatellite regions. MSI status is a prognostic marker and can influence treatment choices, especially concerning the efficacy of checkpoint inhibitor therapies. GOLPH3 showed a positive correlation with the TMB score (cor = 0.104, *p* = 0.033), indicating a link with the overall mutation rate, but not with the MSI score (Figure 2A). Next, we explored potential differences in drug sensitivity between patients with high and low GOLPH3 expression. Patients with elevated GOLPH3 levels were associated with a better response to 5-fluorouracil, gemcitabine, and sunitinib, as shown in Figures 2B–D. Finally, the mutation landscape of GOLPH3 in colon cancer is presented in Figures 2E, F.

### Immune microenvironment analysis

**FIGURE 3:**
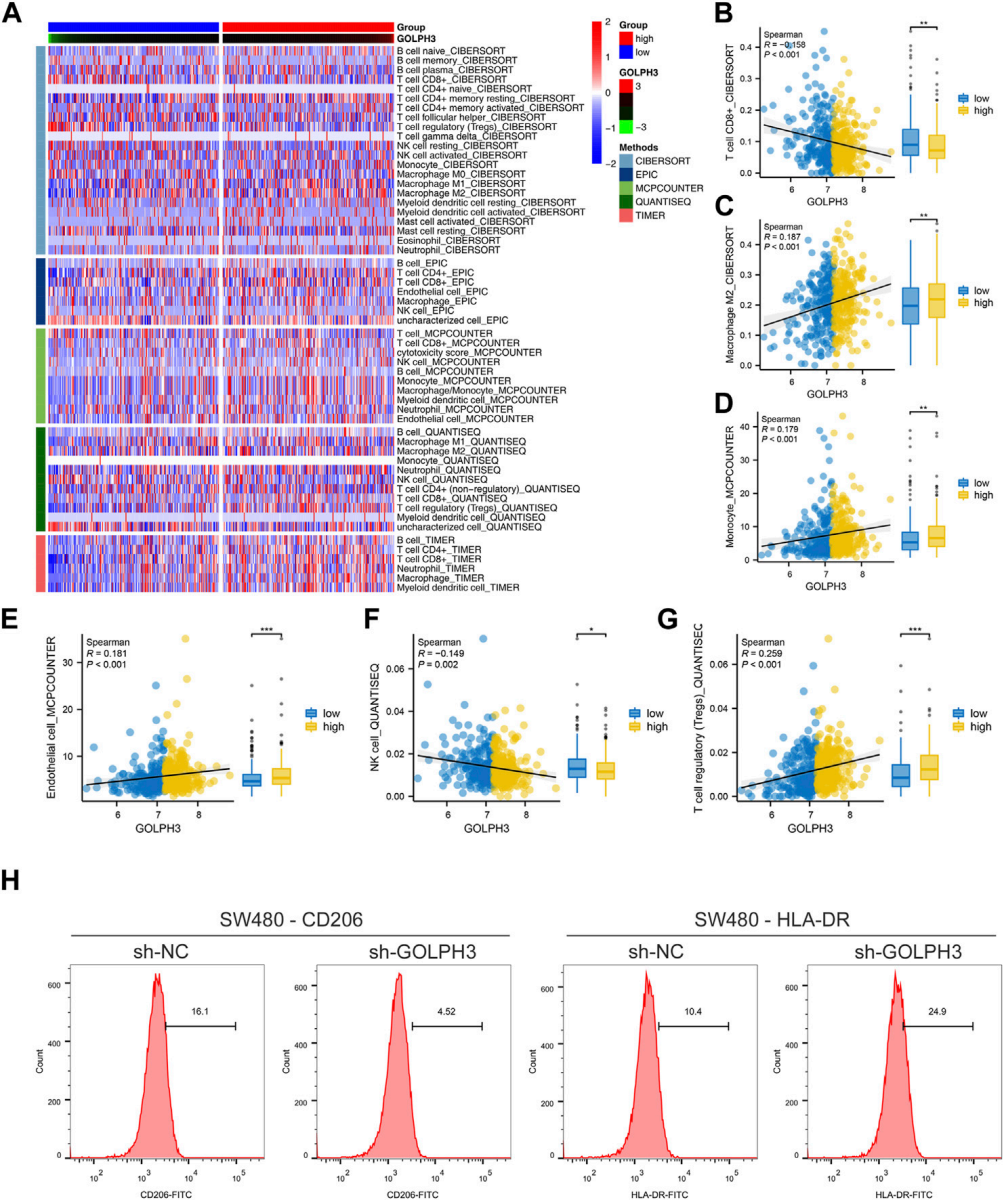
Impact of GOLPH3 on the immune microenvironment of colon cancer. Notes: **(A)** Overview of the cellular composition within the colon cancer microenvironment, highlighting the differential expression levels of GOLPH3 across various cell types. The immune cells was quantified using CIBERSORT, EPIC, MCPCOUNTER, QUANTISEQ and XCELL algorithm; B–G: Correlation analysis between GOLPH3 expression and the abundance of key immune cell types in the colon cancer microenvironment, including M2 macrophages **(B)**, monocytes **(C)**, endothelial cells **(D)**, regulatory T cells (Tregs) **(E)**, CD8^+^ T cells **(F)**, and natural killer (NK) cells **(G)** (Spearman correlation analysis), * = *p* < 0.05, ** = *p* < 0.01, *** = *p* < 0.001; **(H)** Functional assay depicting the effects of conditioned medium from GOLPH3-knockdown SW480 cells on the phenotype of THP-1 derived macrophages. The macrophages exhibited a significant shift towards an M2-like phenotype upon treatment, as evidenced by increased CD206 expression and decreased HLA-DR expression, analyzed via flow cytometry. Statistical significance was determined using Student’s t-test, comparing the treated group to controls.

Tumor progression is frequently influenced by the surrounding tumor microenvironment. Using gene expression data, we investigated the impact of GOLPH3 on this environment within colon cancer (Figure 3A). Our analysis revealed a positive correlation between GOLPH3 expression and the presence of M2 macrophages, monocytes, endothelial cells, and Tregs, while a negative correlation was found with CD8^+^ T cells and NK cells (Figures 3B–G). Furthermore, GOLPH3 showed a positive correlation with immune checkpoints like PDCD1LG2 (Cor = 0.209), CTLA4 (Cor = 0.101) and CD274 (Cor = 0.191) (Supplementary Figure S5A–D). Based on these findings, we hypothesized that GOLPH3 plays a crucial role in communication between cancer cells and tumor-associated macrophages (TAMs). To validate this hypothesis, THP-1 (macrophage) cells were treated with culture supernatant from control and SW480 cells with reduced GOLPH3 expression for 24 h. Compared to the control supernatant, exposure to the GOLPH3-depleted supernatant resulted in a shift of THP-1 cells towards a less immunosuppressive M2 phenotype, characterized by lower CD206 and higher HLA-DR expression (Figure 3H).

### GOLPH3 was upregulated by the BPA exposure

**FIGURE 4:**
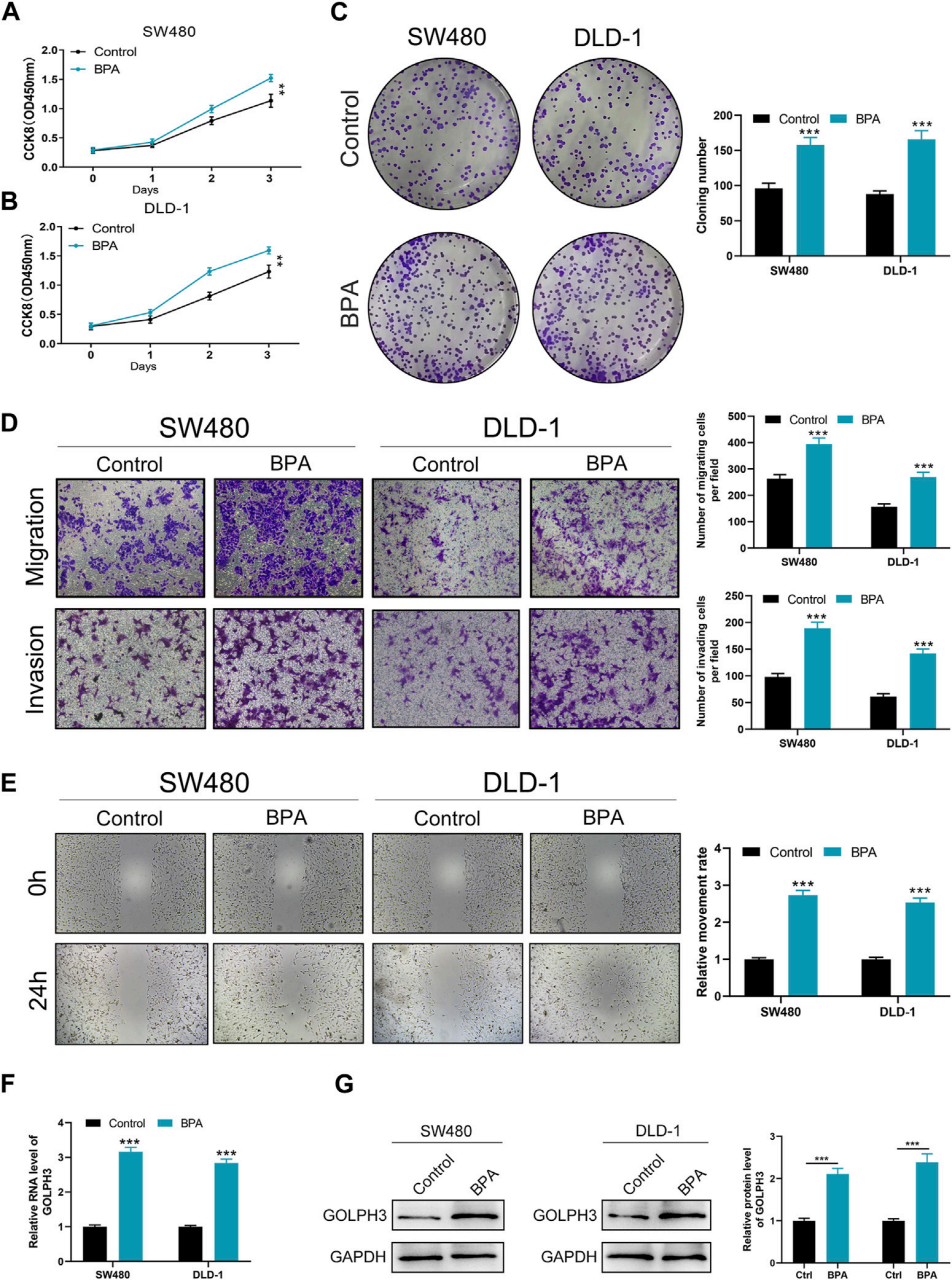
Effects of BPA exposure on the proliferation, migration, and invasion abilities of colon cancer cells. Notes: **(A–C)** Evaluation of cell proliferation following BPA exposure in colon cancer cell lines SW480 and DLD-1, measured by CCK8 **(A, B)** and colony formation assays **(C)**. These panels depict a significant increase in cell proliferation rate and colony formation ability in cells treated with BPA compared to control groups. Statistical analysis was conducted using Student’s t-test, ** = *p* < 0.01, *** = *p* < 0.001; **(D)** Transwell migration and invasion assays demonstrate enhanced migration and invasion capacities of SW480 and DLD-1 cells after BPA exposure. This figure quantitatively presents the number of cells that migrated or invaded through the membrane. The differences were statistically analyzed using an unpaired *t*-test, *** = *p* < 0.001; **(E)** Wound-healing assays show accelerated closure of scratch wounds in BPA-treated colon cancer cells compared to controls. Quantification of wound closure rates provides a direct measurement of cell migration *in vitro*. Statistical significance was evaluated using Student’s t-test, *** = *p* < 0.001; F–G: Analysis of GOLPH3 expression at mRNA **(F)** and protein levels **(G)** in colon cancer cells after BPA exposure, demonstrating upregulation of GOLPH3. These results suggest a mechanism by which BPA may promote cancer progression, potentially through the modulation of GOLPH3 expression. qRT-PCR and Western blot analyses were used for quantification, with statistical differences assessed by Student’s t-test, *** = *p* < 0.001.

**FIGURE 5:**
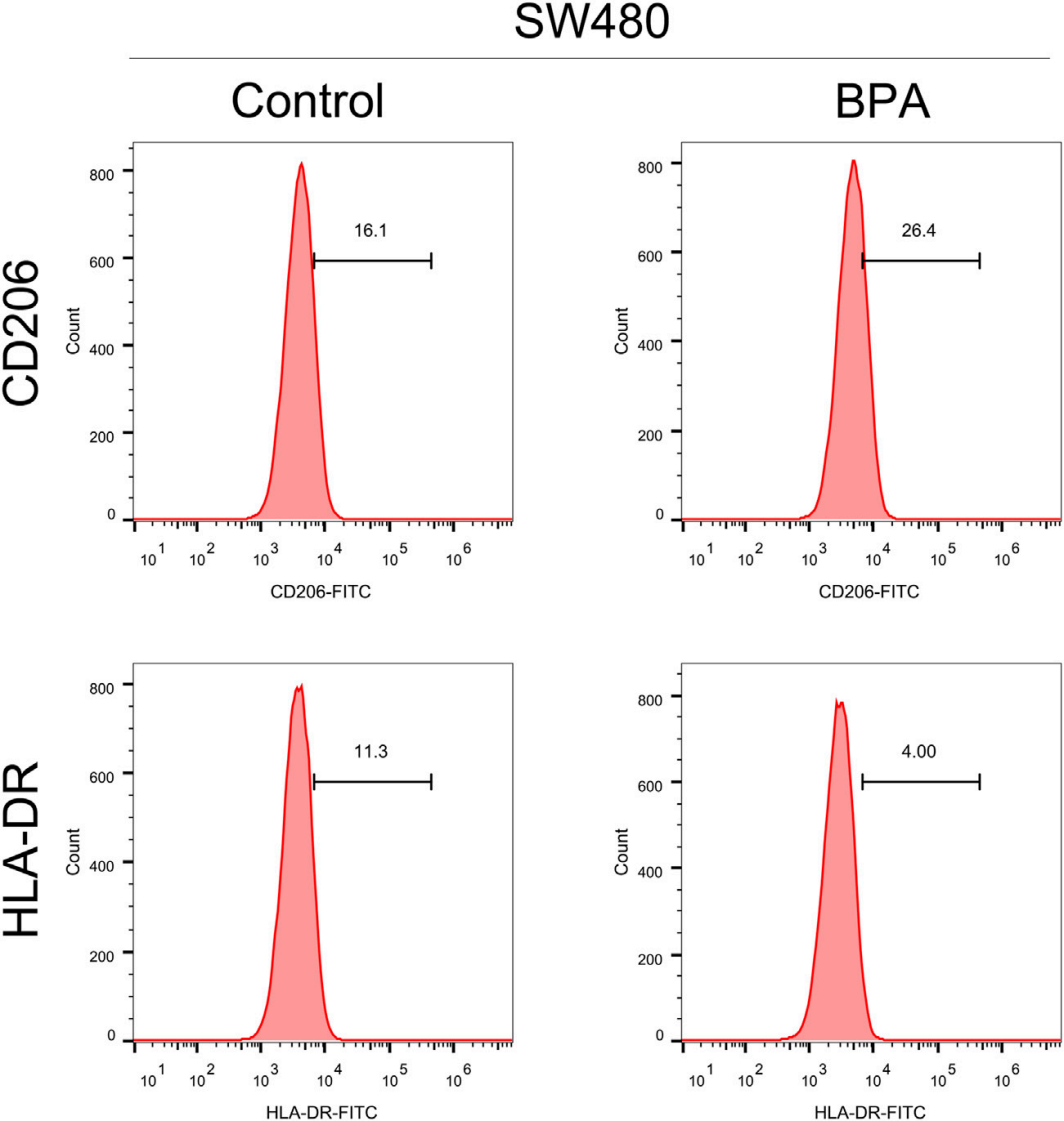
The effect of BPA on the macrophage polarization. Notes: This figure illustrates the effects of exposing THP-1 derived macrophages to 1 μM BPA for 24 h, and the subsequent polarization towards an M2-like phenotype. Post-exposure, macrophages demonstrated increased expression of M2 markers (CD206), indicative of a shift towards an anti-inflammatory state, and decreased expression of M1 markers (HLA-DR). These phenotypic changes were analyzed using flow cytometry. Statistical significance of differences between the BPA-treated group and control group was assessed using Student’s t-test.

**FIGURE 6:**
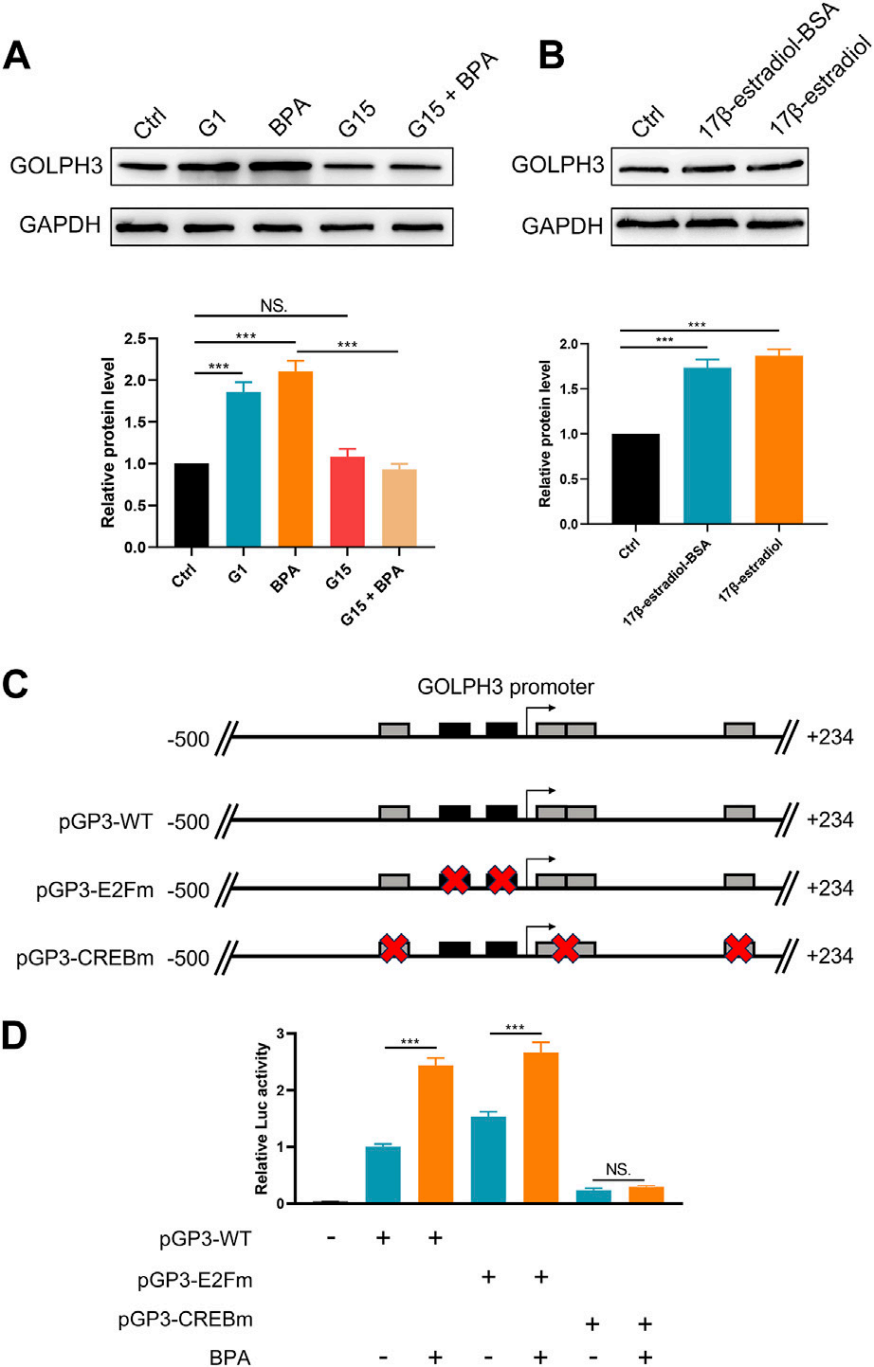
BPA-induced GOLPH3 expression is mediated through GPER and involves CREB/ATF motifs. Notes: **(A)** Western blot analysis showing GOLPH3 protein levels after treatment with G1 (GPER agonist), G15 (GPER antagonist), and BPA in cultured cells. BPA and G1 significantly increase GOLPH3 expression, which is blocked by G15, suggesting GPER involvement, Student’s t-test, *** = *p* < 0.001, NS = *p* > 0.05; **(B)** Comparison of GOLPH3 expression following treatment with 17β-estradiol-BSA (non-permeable) and 17β-estradiol (permeable). Both treatments similarly upregulate GOLPH3, indicating membrane-initiated estrogen signaling, Student’s t-test, *** = *p* < 0.001; **(C)** Luciferase reporter assays in cells transfected with pGP3-WT (wild-type GOLPH3 promoter), pGP3-E2Fm (mutated CREB/ATF motifs), and pGP3-CREBm (mutated E2F motifs); **(D)** Enhanced luciferase activity in pGP3-WT and pGP3-E2Fm with BPA treatment indicates that GOLPH3 regulation is dependent on CREB/ATF motifs, unaffected in the pGP3-CREBm construct, Student’s t-test, *** = *p* < 0.001, NS = *p* > 0.05.

To investigate the effect of BPA on colon cancer progression, we exposed colon cancer cells to BPA and observed a significant increase in their proliferation ability compared to the control group, as demonstrated by CCK8 and colony formation assays (Figures 4A–C). Flow cytometry analysis revealed that BPA exposure also significantly reduced apoptosis rates in SW480 cells (Supplementary Figure S6). Our findings from the Transwell assay demonstrated a significant enhancement in the migratory and invasive capacities of colon cancer cells following exposure to BPA, compared to the control group (Figure 4D). This observation was further corroborated by the wound-healing assay, suggesting a pro-migratory effect of BPA (Figure 4E). Subsequently, we sought to elucidate the potential correlation between GOLPH3 expression and BPA exposure. Intriguingly, we observed that BPA exposure resulted in the upregulation of GOLPH3 mRNA and protein levels in both SW480 and DLD-1 cell lines (Figures 4F,G). Considering that GOLPH3 can be affected by BPA, we further explored the role of BPA in macrophage polarization. Results showed that BPA exposure can also promote M2 polarization of macrophages (Figure 5). Next, we aim to explore the potential mechanisms by which BPA upregulates GOLPH3. Initially, considering the estrogenic properties of BPA, it can initiate downstream signaling cascades through interactions with nuclear or membrane-associated estrogen receptors (ERs or GPER). We used G1 (GPER agonist), G15 (GPER antagonist), 17-beta-estradiol-BSA (observe membrane-limited estrogen effects), 17-beta-estradiol (cell permeability) to treat cells and the protein expression of GOLPH3 was detected. The results showed that exposure to G1 and BPA could significantly increase the protein level of GOLPH3, but G15 completely blocked the effect of BPA (Figure 6A). In addition, both 17β-estradiol-BSA and 17β-estradiol can induce GOLPH3 expression. However, 17β-estradiol-BSA and 17β-estradiol have similar effects on increasing GOLPH3 (Figure 6B). These results indicate that the effect of BPA on GOLPH3 is mainly membrane-initiated. Previous research has demonstrated that the promoter region of GOLPH3 contains response elements for E2F and CREB/ATF (Peñalver-González et al., 2019). BPA can bind to and activate GPER, which in turn may activate E2F and CREB, potentially involved in the regulation of GOLPH3 expression (Ankers et al., 2016; Chen et al., 2016; Gorowska-Wojtowicz et al., 2022; Hu et al., 2023). Based on this, we hypothesize that BPA regulates GOLPH3 expression through GPER-mediated downstream activation of CREB and E2F. To test this hypothesis, we utilized three plasmids in our experiments. As shown in Figure 6C, the pGP3-WT plasmid contains the full GOLPH3 promoter region, including all CREB/ATF and E2F motifs. The pGP3-E2Fm plasmid contains only E2F motifs, with all CREB/ATF motifs mutated. Conversely, the pGP3-CREBm plasmid contains only CREB/ATF motifs, with all E2F motifs mutated. The results indicate that BPA exposure significantly increases luciferase activity in pGP3-WT and pGP3-E2Fm, but does not affect the activity in pGP3-CREBm (Figure 6D). Therefore, we believe that the impact of BPA on GOLPH3 is controlled by the CREB/ATF motifs in its promoter region.

### BPA promotes colon cancer proliferation, invasion and migration through upregulating GOLPH3

**FIGURE 7:**
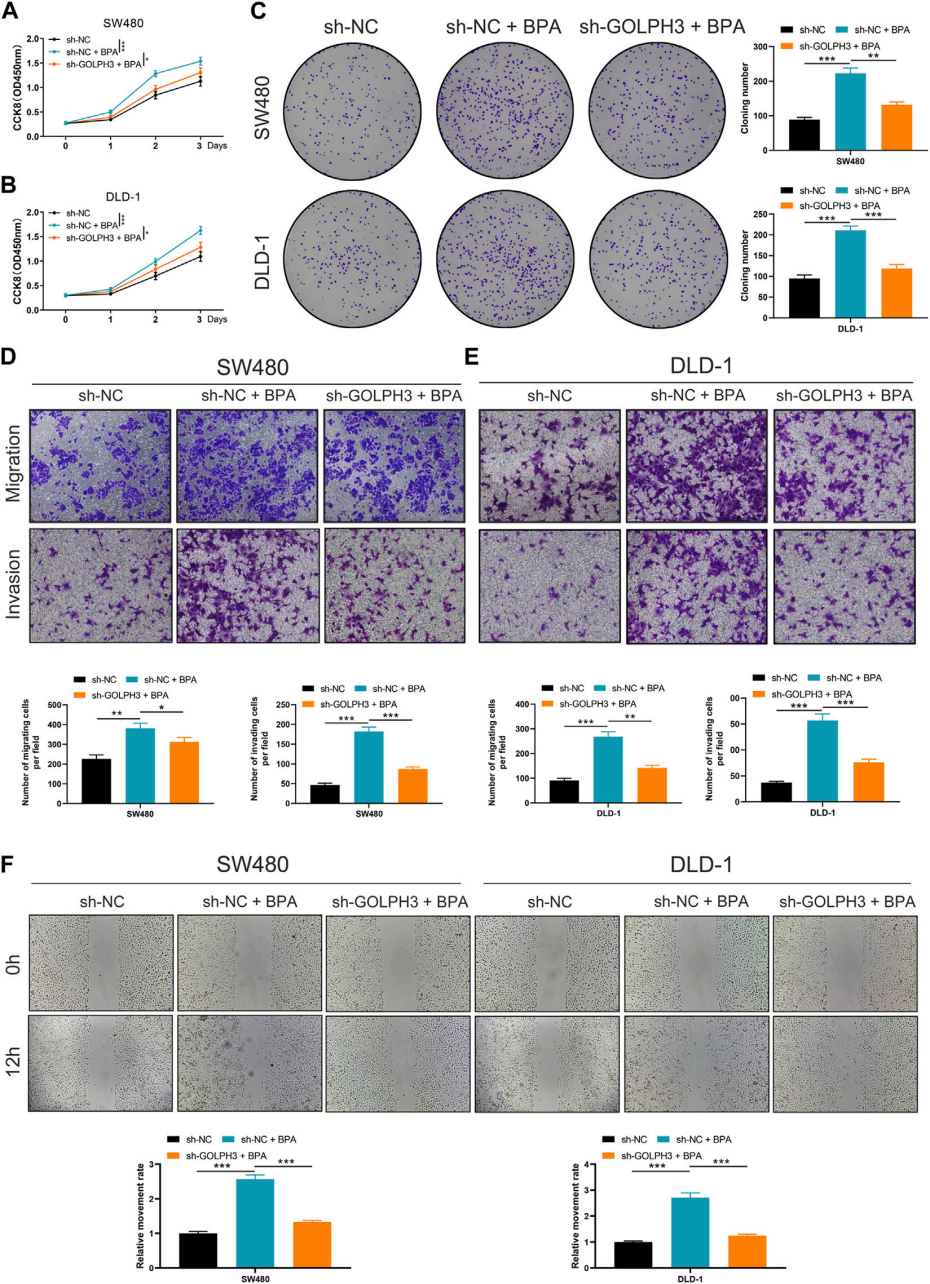
Impact of GOLPH3 knockdown on BPA-induced proliferation, migration, and invasion of colon cancer cells. Notes: **(A–C)** Examination of cell proliferation abilities in colon cancer cell lines (SW480 and DLD-1) post GOLPH3 knockdown and subsequent BPA exposure, assessed using CCK8 **(A, B)** and colony formation assays **(C)**. These sections illustrate a significant reduction in BPA-induced proliferation upon GOLPH3 suppression, highlighting GOLPH3’s role in mediating BPA’s pro-proliferative effects. Statistical significance was determined through Mann-Whitney U test, * = *p* < 0.05, ** = *p* < 0.01, *** = *p* < 0.001; **(D, E)** Transwell assays demonstrate decreased migration and invasion capabilities in GOLPH3-knockdown cells treated with BPA, compared to cells treated with BPA alone. This suggests GOLPH3’s involvement in BPA-enhanced cell motility. Data are represented as the number of migrated or invaded cells, with statistical analysis performed using Mann-Whitney U test, * = *p* < 0.05, ** = *p* < 0.01, *** = *p* < 0.001; **(F)** Wound-healing assays further corroborate the transwell assay findings by showing slower closure rates in scratch wounds for cells with GOLPH3 knockdown and BPA treatment, as opposed to BPA treatment alone. Quantification of wound closure percentage over time provides a direct measure of cell migration, with statistical differences assessed using Mann-Whitney U test, *** = *p* < 0.001.

**FIGURE 8:**
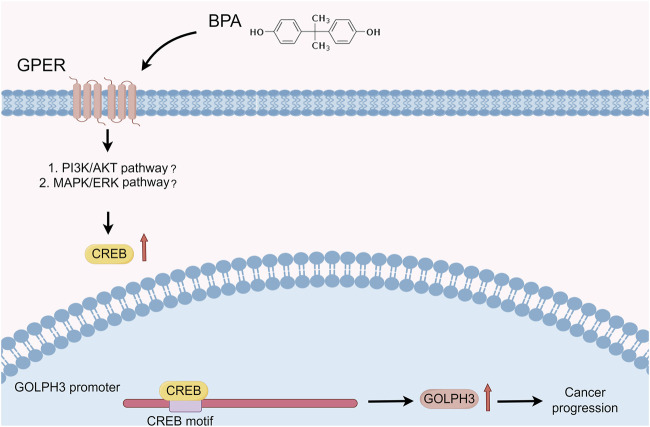
Proposed mechanism of BPA-mediated regulation of GOLPH3 via the GPER signaling pathway. Notes: This schematic illustrates the interaction between BPA and GPER, leading to downstream activation of signaling pathways that modulate GOLPH3 expression. Key components include (Kiess and Haeusler, 2021): BPA binding to GPER, located on the cell membrane (Yilmaz et al., 2020); subsequent activation of potential downstream signaling cascades, including MAPK/ERK and PI3K/Akt pathways (Ribeiro et al., 2017); translocation of transcription factors into the nucleus, resulting in enhanced GOLPH3 transcription.

To investigate the functional role of this upregulation, we examined whether BPA could promote cancer progression via GOLPH3 upregulation. The CCK8 and colony formation assays revealed that BPA treatment enhanced the proliferative capacity of colon cancer cells, and this effect was significantly attenuated by GOLPH3 knockdown (Figures 7A–C). Furthermore, Transwell and wound-healing assays consistently demonstrated a similar abrogation of BPA-induced migration and invasion upon GOLPH3 knockdown (Figures 7D–F). The proposed mechanism of BPA-mediated regulation of GOLPH3 through the GPER signaling pathway is shown in Figure 8. We further developed a nomogram to improve the predictive capability of GOLPH3 during clinical practice (Supplementary Figure S7).

## Discussion

Colorectal cancer is a leading cause of cancer-related deaths worldwide, highlighting the importance of early detection and treatment (Berlau et al., 2004). Recent research points to a complex interplay between genetic, lifestyle, and environmental factors in colorectal cancer development, with environmental influences gaining increasing attention (O'Keefe, 2016). EDCs such as BPA have emerged as a public health concern due to their widespread presence and potential to disrupt hormonal systems (Nair et al., 2022). This study sought to shed light on how BPA contributes to colorectal cancer pathogenesis. We focused on the interaction between BPA and key molecular pathways, such as those involving the GOLPH3 gene. By examining these interactions, we aimed to uncover mechanisms by which BPA could exacerbate cancer traits, underscoring the urgency for strategies to minimize BPA exposure and its associated health risks.

This study represents the first investigation, to our knowledge, into the link between BPA and the GOLPH3 gene in colon cancer. Our findings suggest a potential pathway where BPA exposure may influence colon cancer progression through GOLPH3 upregulation, associated with enhanced malignant characteristics in colon cancer cells observed in our *in vitro* analyses. However, caution is warranted when interpreting these results due to their *in vitro* nature. Supporting the potential significance of GOLPH3 in tumorigenesis, we observed elevated protein levels in colon cancer tissues compared to normal tissues, and its presence across various cell types within the tumor microenvironment. Furthermore, our exploration into GOLPH3’s involvement in critical oncogenic pathways, such as the G2M checkpoint, strengthens the possibility of its impact on cancer progression.

BPA, a common chemical found in many consumer products, has raised concerns in recent years due to its potential impact on human health, particularly cancer development (Murata and Kang, 2018). Studies suggest BPA may promote cancer by mimicking estrogen, disrupting hormone function and inducing DNA damage. This potential carcinogenicity highlights the need to limit exposure and further investigate its health effects. For instance, Medellín-Garibay et al. (Medellín-Garibay et al., 2023). linked abnormal urinary BPA levels in women with cervical cancer, suggesting a possible link between exposure and the disease. Additionally, Palacios-Arreola et al. (Palacios-Arreola et al., 2022). observed that BPA exposure in mice models altered the immune microenvironment of breast cancer and promoted lung metastasis. GOLPH3, a recently discovered molecule, has emerged as a player in cancer progression (Giansanti and Piergentili, 2022). Multiple studies have reported the upregulation of GOLPH3 in various cancers, strongly associating it with tumor advancement, metastasis, and patient prognosis (Kuna and Field, 2019; Song et al., 2021). GOLPH3 is believed to promote cancer cell growth, inhibit cell death, and enhance migration through the regulation of signaling pathways like AKT/mTOR (Yu et al., 2020). These findings position GOLPH3 as a promising target for cancer therapy, offering novel avenues for treatment (Giansanti and Piergentili, 2022). Our study contributes to a comprehensive understanding of GOLPH3’s role in colon cancer, particularly its network of effects.

To mimic real-world exposure scenarios in a controlled setting, we selected a BPA concentration of 1 μM for 24 h, which aligns with the average human plasma detection level of BPA (0.67 μg/L). This approach facilitated the investigation of BPA’s effects on cellular processes relevant to human health. Under these conditions, we observed BPA-induced upregulation of GOLPH3 expression. As an endocrine disruptor, BPA mimics estrogen structure and function, potentially interacting with ERα, ERβ and the GPER (Buoso et al., 2020a; Masi et al., 2021; Wang et al., 2021). This interaction could activate signaling pathways like PI3K/AKT and ERK/MAPK, known to be involved in cell proliferation, survival, and migration (Sun et al., 2024). Activation of these pathways could lead to transcriptional and post-transcriptional modifications that upregulate GOLPH3 (Buoso et al., 2020b). GOLPH3, in turn, has been implicated in vesicle trafficking and signal transduction, essential for cancer cell proliferation and metastasis. The involvement of GPER suggests a role for rapid, non-genomic signaling by BPA, independent of direct DNA interactions. Additionally, BPA’s binding to estrogen receptors can modulate gene expression through genomic mechanisms involving estrogen response elements (EREs) in DNA (Hafezi and Abdel-Rahman, 2019). This dual mode of action, both genomic and non-genomic, highlights the complex interplay between BPA and estrogen receptor signaling, ultimately leading to GOLPH3 upregulation and contributing to the tumorigenic potential of colon cancer cells.

Our findings also revealed GOLPH3’s involvement in multiple oncogenic pathways, particularly those related to cell cycle progression, DNA replication, and TGF-β signaling. Cell cycle regulation, a fundamental process tightly linked to cancer development, is significantly impacted by GOLPH3 participation (Schafer, 1998). Furthermore, GOLPH3 is associated with DNA replication, another crucial step in cancer cell proliferation (Ekundayo and Bleichert, 2019). The enrichment of GOLPH3 within the TGF-β signaling pathway is particularly intriguing, as this pathway is well-established as a promoter of tumor growth and metastasis in various cancers (Tzavlaki and Moustakas, 2020). The G2M checkpoint, a critical stage in the cell cycle that ensures cells are prepared for mitosis (Smith et al., 2020), also involves GOLPH3. This involvement suggests a potential link to the ability of cancer cells to evade cell cycle arrest and drug therapy. Importantly, our study also addresses the pharmacological implications of GOLPH3 expression. We observed that higher GOLPH3 expression correlates with lower IC50 values for drugs such as 5-fluorouracil, gemcitabine, and sunitinib, suggesting a better pharmacological response in these patients. This observation highlights a dual role of GOLPH3—not only as an oncogene promoting tumor progression but also in modulating drug sensitivity. These effects of GOLPH3 on drug response may be mediated through its influence on Golgi apparatus composition and trafficking functions, which can affect the cellular uptake and distribution of chemotherapeutic drugs (Kuna and Field, 2019). Moreover, GOLPH3 may impact drug resistance mechanisms by altering vesicular trafficking of drug efflux pumps, regulating apoptosis which is a target of many chemotherapeutic agents, and modifying cellular responses to oxidative stress induced by chemotherapy (Li et al., 2014; Hong et al., 2020; Giansanti and Piergentili, 2022). These insights provide a crucial understanding of how GOLPH3 could be targeted or considered in therapeutic strategies.

The correlation between GOLPH3 expression and the tumor microenvironment in colon cancer is highly significant. It has been reported that GOLPH3 is positively correlated with the presence of M2 macrophages, monocytes, endothelial cells, and Tregs, but negatively correlated with CD8^+^ T cells and NK cells. M2 macrophages promote tumor development, spread, and suppress immune responses (Locati et al., 2020). Similarly, Tregs can reportedly suppress anti-tumor immune responses (Tie et al., 2022). Endothelial cells contribute to tumor angiogenesis, supplying nutrients for tumor growth (Verhoeven et al., 2021). Conversely, CD8^+^ T cells and NK cells are the main cytotoxic immune cells in the tumor microenvironment, crucial for inhibiting tumor growth and metastasis (van der Leun et al., 2020). The presence of GOLPH3 in these specific cell types suggests a critical role in regulating the tumor microenvironment, potentially facilitating colon cancer growth and progression. Furthermore, a study demonstrating the pro-inactivating immune functions of BPA in monocytes, along with the established link between BPA and GOLPH3, provides clues for understanding the immune effects of BPA (Buoso et al., 2021). In our study, we identified that GOLPH3 can be modulated by the BPA/GPER axis, which partly elucidates the impact of BPA and GPER on the tumor microenvironment. As a member of the membrane-bound estrogen receptors, GPER initiates a cascade of downstream signaling events that manifest a variety of biological functions (Masi et al., 2021). EDCs are known to alter hormonal communication within the tumor microenvironment. This alteration facilitates a dynamic interaction among tumor cells, stromal cells, and immune cells, which contributes to changes in the extracellular matrix that support tumor development, enhance invasion capabilities, and promote metastasis (Buoso et al., 2020a). The interaction between EDCs and the tumor microenvironment is, to some extent, regulated by hormone receptors such as GPER, which modulate the effects of EDCs on the cellular and molecular composition of the tumor microenvironment (Nowak et al., 2019). Furthermore, the influence of GOLPH3 on the microenvironment may help to explain the impact of GOLPH3-associated EDCs, such as vinclozolin and ethynyl estradiol, on various internal microenvironments of diseases (Boverhof et al., 2008; Skinner et al., 2008; Skinner et al., 2012). Also, some EDCs have been reported to affect the activation of immune cells such as THP-1 monocytes, and our findings could provide a potential mechanistic explanation for these effects. Importantly, given the complex interactions and significant implications of EDCs like ethynyl estradiol in immune modulation, further investigations are warranted to dissect their roles more comprehensively in colorectal cancer within the context of the GPER-GOLPH3 axis (Maddalon et al., 2022; Masi et al., 2022).

Our study acknowledges limitations that require contextualization within the broader BPA, GOLPH3, and colon cancer research landscape. First, reliance on TCGA data introduces potential biases related to demographics and sample size, limiting generalizability to diverse populations. Second, the inherent challenges of bioinformatic research, such as computational models and parameter settings, can affect the interpretability and reliability of our conclusions. These factors necessitate caution when interpreting our findings and highlight the need for future experimental validation to solidify our preliminary insights into the BPA-GOLPH3 axis and its role in colon cancer pathogenesis.

## Data Availability

The raw data supporting the conclusion of this article will be made available by the authors, without undue reservation.

## Supplementary material

SUPPLEMENTARY FIGURE S1 The flow chart of the whole study.

SUPPLEMENTARY FIGURE S2 The upregulated GOLPH3 promote the proliferation, invasion and migration ability of colon cancer cells. Notes: **(A)** qRT-PCR assay indicated a significant upregulation of GOLPH3 in SW480 and DLD-1 cells compared with the normal NCM460 cells (Mann-Whitney U test), *** = P < 0.001; **(B,C)** qRT-PCR was used to detect the expression difference in control and cell transfected with GOLPH3 knockdown plasmid (Mann-Whitney U test) and the sh-GOLPH3#2 has the best knockdown efficiency, * = P < 0.05, *** = P < 0.001; **(D,E)** CCK8 assay was used to detect the proliferation ability of control and colon cancer cells transfected with GOLPH3 knockdown plasmid (Mann-Whitney U test), ** = P < 0.01; **(F)** Colony formation assay was used to detect the proliferation ability of control and colon cancer cells transfected with GOLPH3 knockdown plasmid (Mann-Whitney U test), *** = P < 0.001; **(G)** Transwell assay was used to detect the invasion and migration ability of control and colon cancer cells transfected with GOLPH3 knockdown plasmid (Mann-Whitney U test), *** = P < 0.001; **(H)** Wound-healing assay was used to detect the migration ability of control and colon cancer cells transfected with GOLPH3 knockdown plasmid (Mann-Whitney U test), ** = P < 0.01.

SUPPLEMENTARY FIGURE S3 Effects of GOLPH3 overexpression in SW480 cells Notes: **(A)** qRT-PCR results showing GOLPH3 mRNA levels significantly increased in SW480 cells after GOLPH3 overexpression compared to the vector control group, (Mann-Whitney U test), *** = P < 0.001; **(B,C)** Cell proliferation assessed by CCK8 and colony formation assays, demonstrating enhanced proliferative capabilities in GOLPH3-overexpressing cells (Mann-Whitney U test), *** = P < 0.001; **(D,E)** Increased invasion and migration abilities in GOLPH3-overexpressing SW480 cells, as evidenced by transwell and wound-healing assays, respectively (Mann-Whitney U test), *** = P < 0.001.

SUPPLEMENTARY FIGURE S4 Comprehensive analysis of GOLPH3 expression patterns in colon cancer compared to normal colon tissue Notes: **(A)** Quantitative analysis of GOLPH3 mRNA expression levels across various human organs, highlighting its ubiquitous presence and suggesting its broad biological significance; **(B,C)** Comparison of GOLPH3 mRNA expression levels in colon cancer tissues versus matched normal colon tissues **(B)**, and in a cohort of colon cancer tissues without matched normals **(C)**. The analysis demonstrates a statistically non-significant difference in mRNA levels, suggesting post-transcriptional regulation might play a role in GOLPH3 expression in cancerous tissues. Statistical analysis was performed using a paired t-test for **(B)** and an unpaired t-test for **(C)**, with p-values reported directly in the figures; **(D,E)** Immunohistochemistry analysis showing the protein expression levels of GOLPH3 in normal colon tissue **(D)** and colon cancer tissue **(E)**. An evident upregulation of GOLPH3 protein in colon cancer tissues suggests a potential post-transcriptional regulatory mechanism affecting its expression; **(F–K)** Single-cell RNA sequencing data analysis from multiple publicly available databases, including EMTAB8107, GSE108989, GSE139555, GSE146771_10X, GSE136394, and GSE146771, illustrates the expression profile of GOLPH3 across different cell types in the colon cancer microenvironment.

SUPPLEMENTARY FIGURE S5 Correlation between GOLPH3 and immune checkpoints Notes: **(A–D)** Association between GOLPH3 expression and critical immune checkpoints, including PDCD1LG2 (PD-L2) **(A)**, PDCD1 **(B)**, CTLA4 **(C)**, and CD274 (PD-L1) **(D)** (Spearman correlation analysis).

SUPPLEMENTARY FIGURE S6 BPA exposure and apoptosis in SW480 cells.

SUPPLEMENTARY FIGURE S7 GOLPH3 predictive nomogram for clinical outcome.
