# Supplementary figures and images for: Upregulation of GOLPH3 mediated by Bisphenol a promotes colorectal cancer proliferation and migration: evidence based on integrated analysis
Source: Front Pharmacol. 2024 May 17;15:1337883. doi: 10.3389/fphar.2024.1337883 (PMC11143881; doi:10.3389/fphar.2024.1337883)

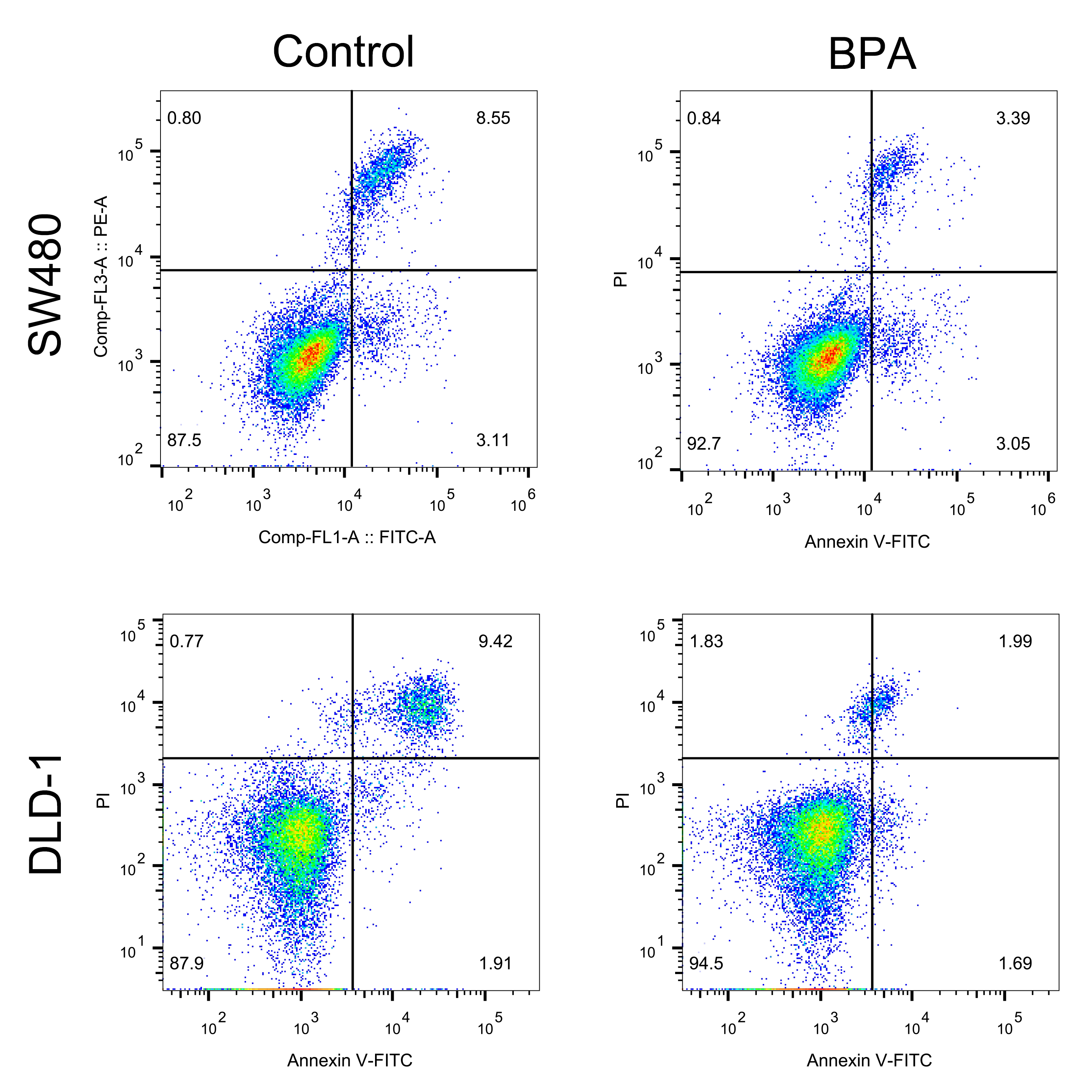

Supplement: Supplementary file 1 [file Image6.TIF]

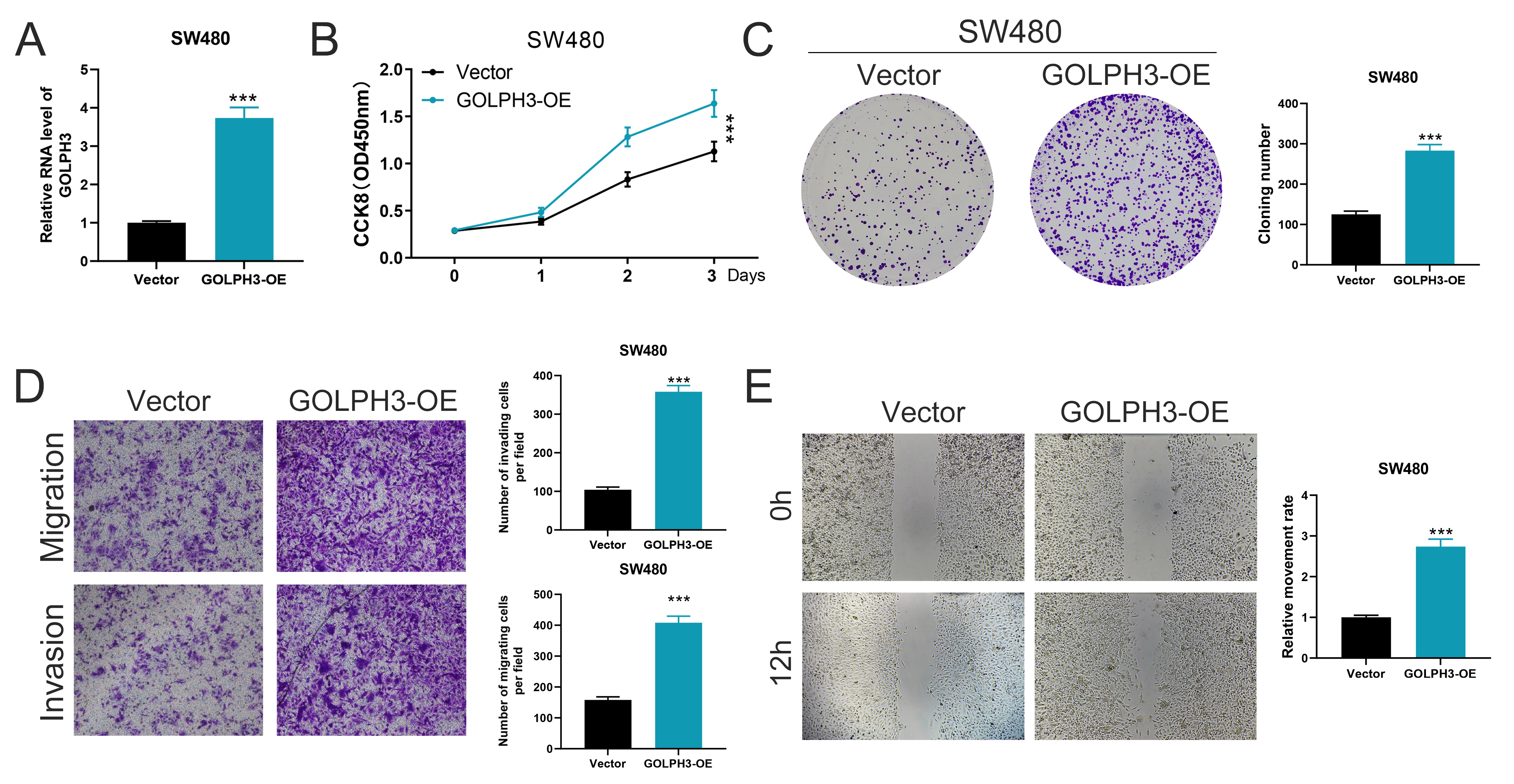

Supplement: Supplementary file 2 [file Image3.TIF]

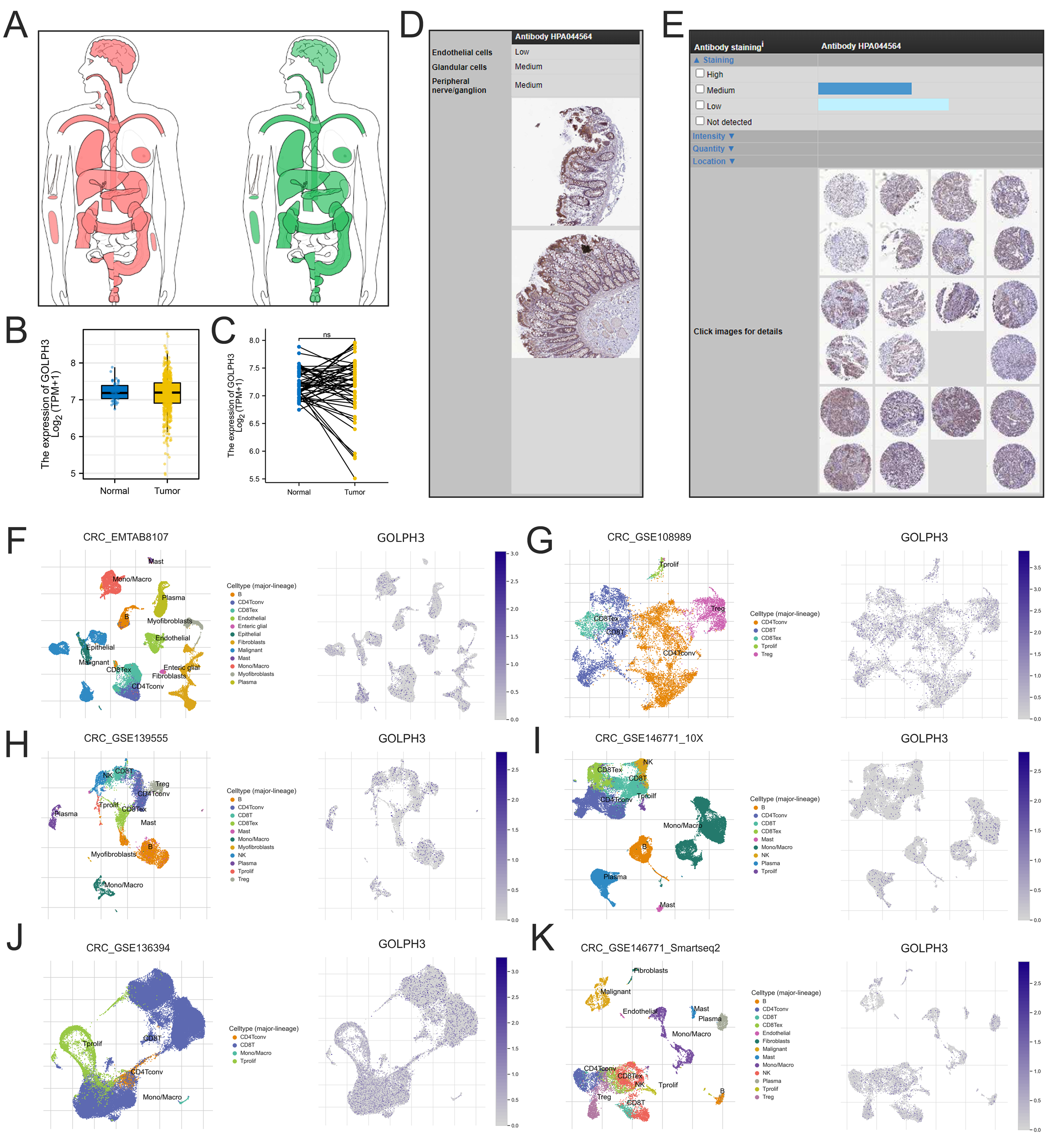

Supplement: Supplementary file 3 [file Image4.TIF]

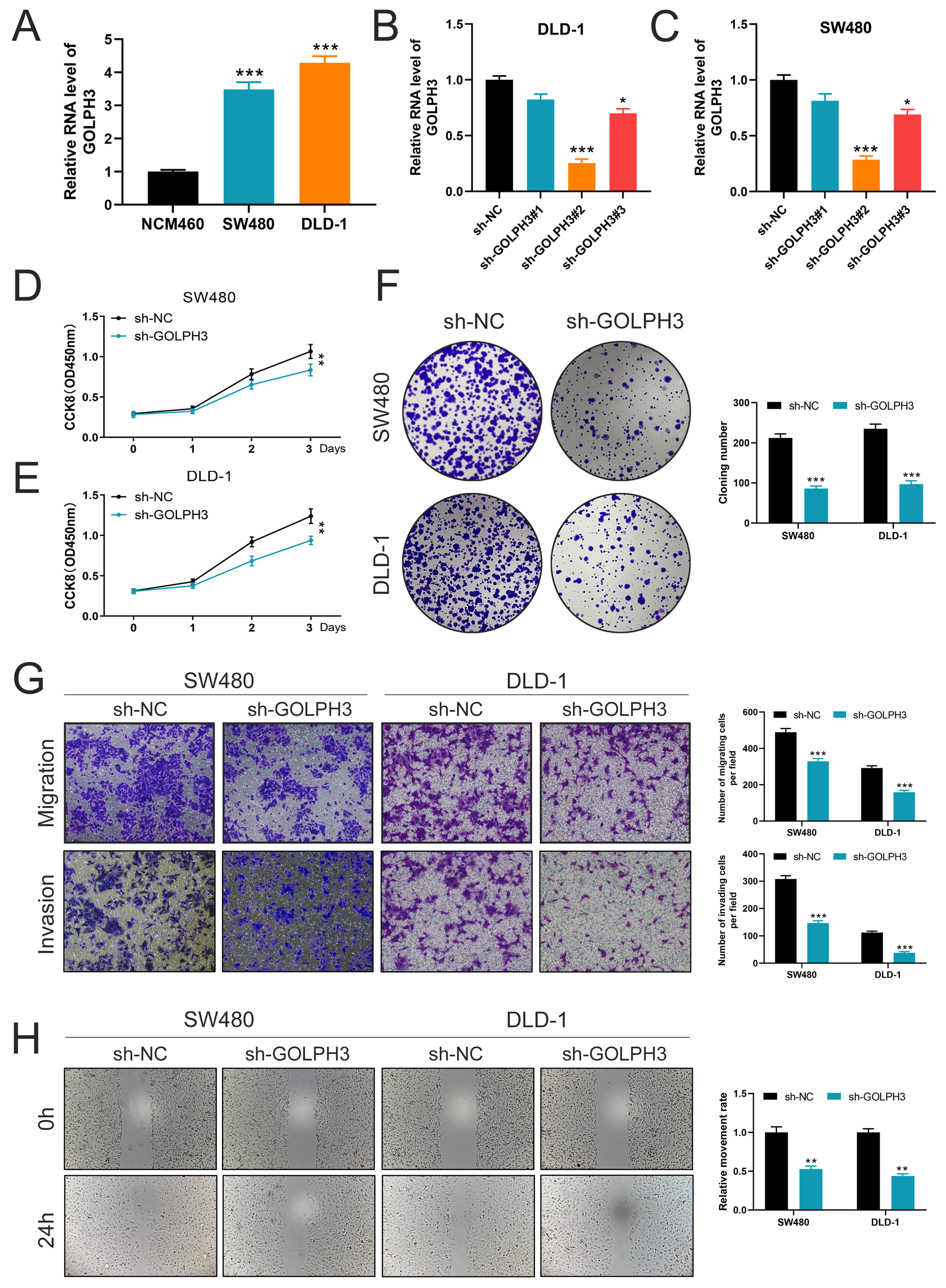

Supplement: Supplementary file 4 [file Image2.TIF]

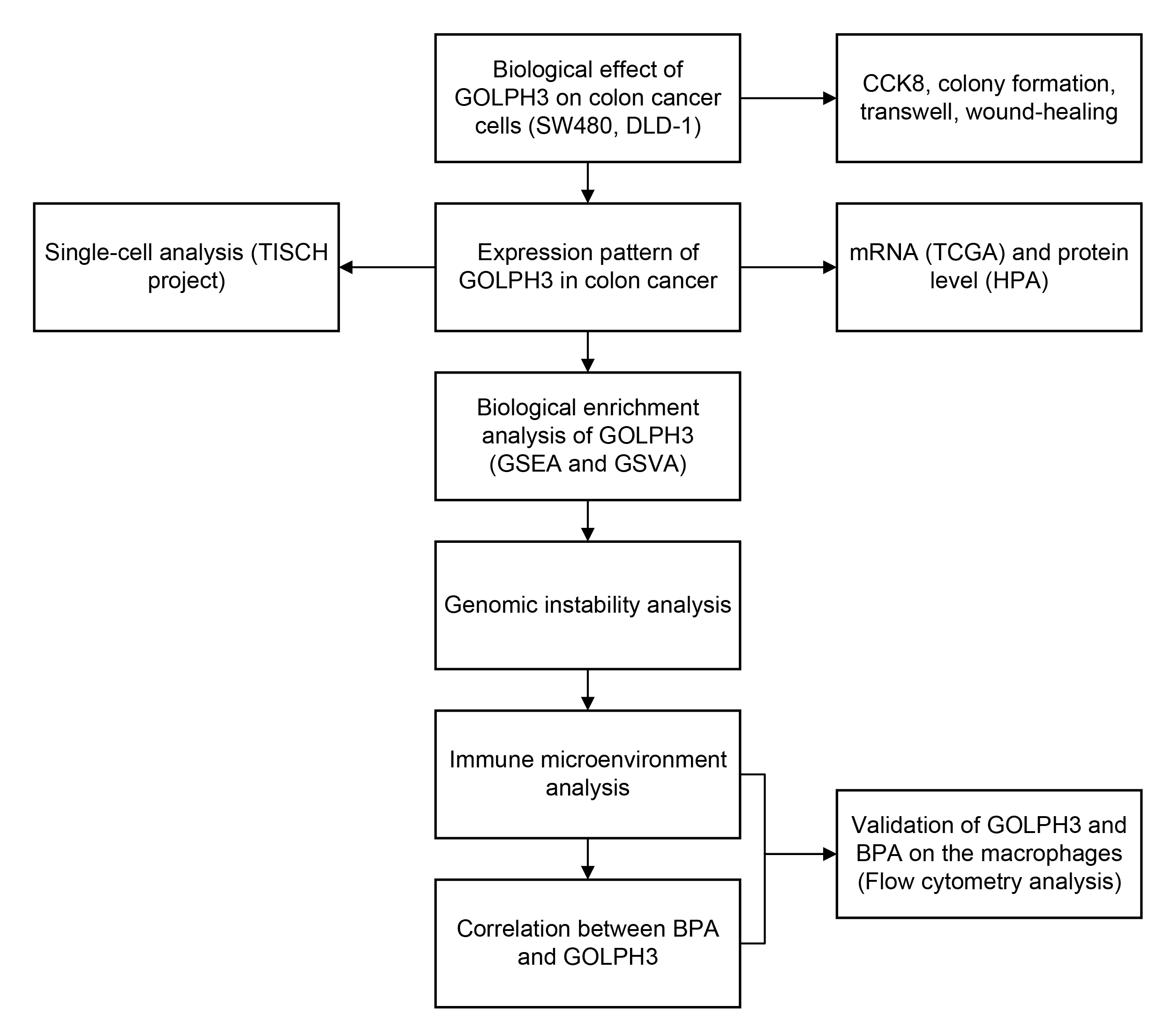

Supplement: Supplementary file 5 [file Image1.TIF]

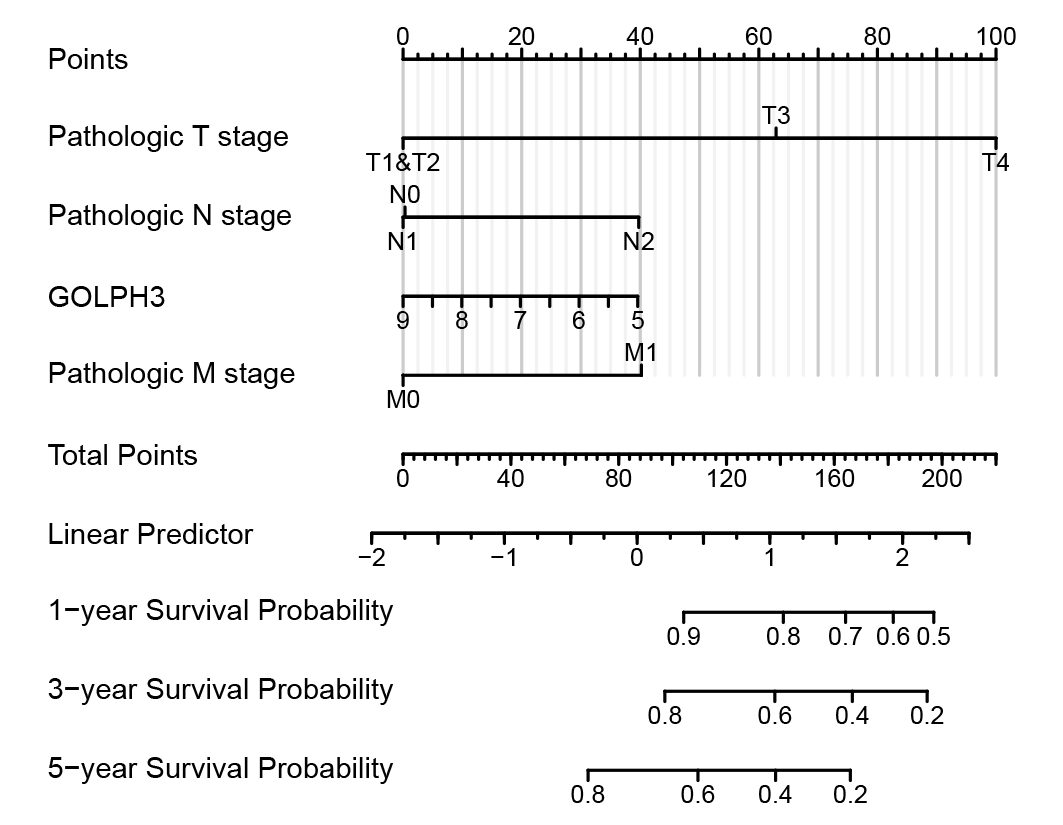

Supplement: Supplementary file 6 [file Image7.TIF]

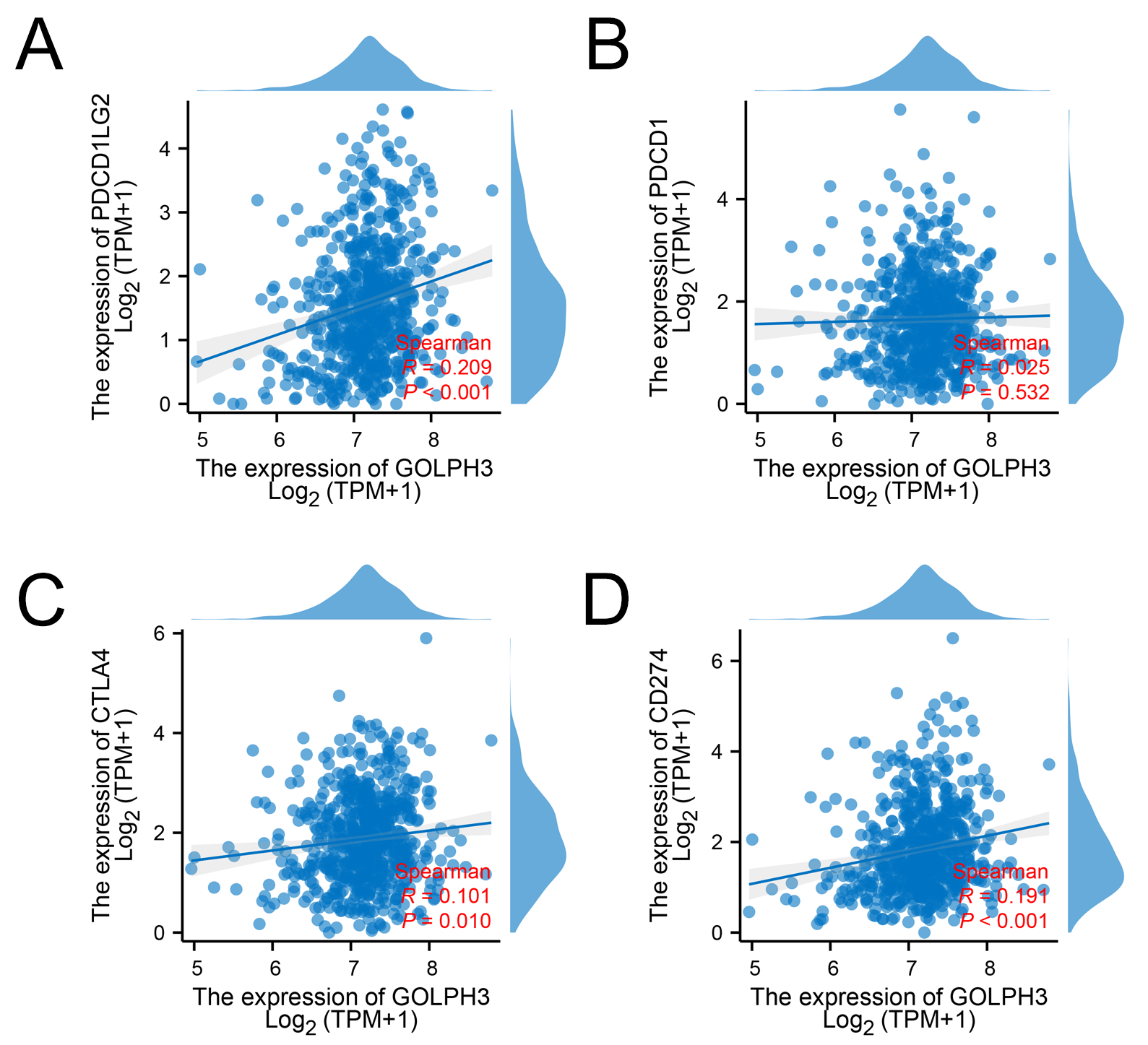

Supplement: Supplementary file 7 [file Image5.TIF]
